# Caudiquinol: A Meroterpenoid with an Intact C20 Geranylgeranyl Chain Isolated from *Garcinia caudiculata*

**DOI:** 10.3390/molecules29153613

**Published:** 2024-07-31

**Authors:** Maya Valmiki, Stephen Ping Teo, Pedro Ernesto de Resende, Simon Gibbons, A. Ganesan

**Affiliations:** 1 School of Pharmacy, University of East Anglia, Norwich Research Park, Norwich NR4 7TJ, UK; m.valmiki@uea.ac.uk; 2 Forest Department Sarawak Headquarters, Medan Raya, Petra Jaya, Kuching 93050, Sarawak, Malaysia; stephetp@sarawak.gov.my; 3 The John Innes Centre, Norwich Research Park, Norwich NR4 7UH, UK; pedro.de-resende@jic.ac.uk; 4 Natural and Medical Sciences Research Center, University of Nizwa, PC 616, Birkat Al-Mauz, Nizwa P.O. Box 33, Oman; simon@unizwa.edu.om

**Keywords:** natural products, meroterpenoids, hydroquinones, *Garcinia* species

## Abstract

The tropical *Garcinia* genus of flowering plants is a prolific producer of aromatic natural products including polyphenols, flavonoids, and xanthones. In this study, we report the first phytochemical investigation of *Garcinia caudiculata* Ridl. from the island of Borneo. Fractionation, purification, and structure elucidation by MS and NMR resulted in the discovery of two meroterpenoids. One was a benzofuranone lactone previously isolated from *Iryanthera grandis* and *Rhus chinensis*, and the second was a new hydroquinone methyl ester that we named caudiquinol. Both natural products are rare examples of plant meroterpenoids with an intact geranylgeranyl chain.

## 1. Introduction

**Figure 1:**
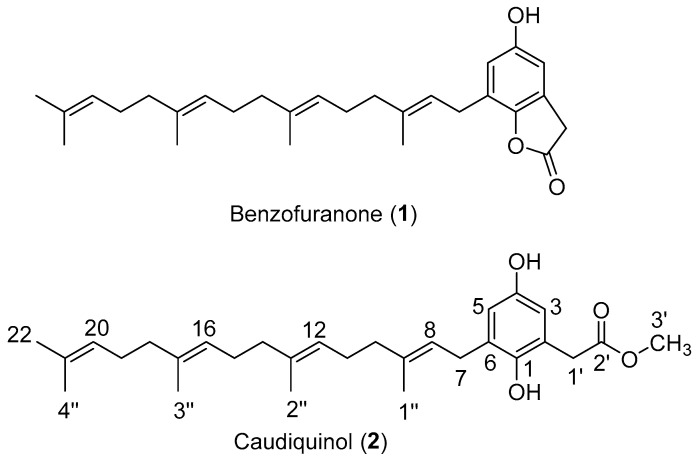
Benzofuranone (**1**) and caudiquinol (**2**), geranylgeranyl meroterpenoids isolated from *Garcinia caudiculata*.

The *Garcinia* genus of saptrees within the Clusiaceae family comprises several hundred species of flowering shrubs and trees that are widely distributed in tropical regions around the world [1]. In addition to producing edible fruit, such as the mangosteen from *Garcinia mangostana*, *Garcinia* species are a prolific source of biologically active secondary metabolites, including polyphenols, flavonoids, and xanthones [2,3,4]. Within this genus, Ridley classified the bunau tree found on the island of Borneo as a new species, *G. caudiculata*, nearly a century ago [5]. However, no phytochemical investigations have appeared until the present work, where we report two meroterpenoids with a geranylgeranyl sidechain: the benzofuranone lactone **1** (Figure 1), previously isolated from two other plant genera, and a new quinol **2** that was given the name caudiquinol.

## 2. Results

Leaves of *G. caudiculata* Ridl. were collected from Lundu, Sarawak, Malaysia, and air-dried before being ground into a powder and extracted with dichloromethane. In a minimum inhibitory concentration (MIC) antibacterial assay, the crude extract was active against methicillin-susceptible *Staphylococcus aureus* (MSSA) 25923 at a level of 128 μg/mL. A portion of this extract of 5 g was subjected to vacuum liquid chromatography (VLC), eluting with a gradient of hexane/ethyl acetate (100:0 to 0:100) to provide 16 fractions. Upon evaporation, the most abundant yellow fractions 10 and 11, each containing ~0.5 g of residue, were selected for further purification. By recycling preparative HPLC, we ultimately obtained 7 mg of **1** from fraction 10 and 6 mg of **2** from fraction 11. Based on the spectroscopic and mass spectrometric data (Supporting Information), we assigned **1** as a benzofuranone lactone (Figure 1) with a C20 geranylgeranyl sidechain. This lactone was first identified in *Iryanthera grandis* of the Myristicaceae family [6] and later, in *Rhus chinensis* of the Anacardiaceae family [7]. It was the subject of a recent total synthesis due to its anti-HIV activity [8].

**Figure 2:**
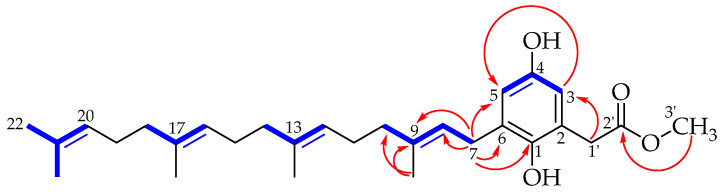
Observed COSY (blue; bold) and HMBC (red arrows) correlations in caudiquinol.

Compound **2** was isolated as a yellow oil with IR absorptions at 3387 and 1714 cm^−1^ suggesting the presence of OH and C=O functional groups. The ^1^H and ^13^C NMR chemical shifts of **2** (Table 1) indicated a carbonyl group at δ_C_ 173.9, a tetrasubstituted aromatic benzene ring with two proton signals in a meta relationship at δ_H_ 6.50 and 6.41 (*J* = 3 Hz), and an unsaturated terpenoid chain with four double bonds and five methyl groups at δ_C_ 16.2, 16.2, 16.3, 17.8, and 25.6. All these features were common to both **1** and **2**. However, **2** uniquely contained a singlet at δ_H_ 3.66 (3H) that correlated with a signal at δ_C_ 52.5. Furthermore, the pseudo-molecular ion of m/z 455.316 observed in the positive-mode ESI MS of **2** was higher than that of **1** by 32 Da. We concluded that the two natural products differed by the addition of a methoxy group. Since the geranylgeranyl moiety and the two aromatic protons within **1** were preserved in **2**, we deduced that the methoxy group was attached as either a phenolic ether or as an ester of the ring-opened lactone. In the HMBC spectrum (Supporting Information), an absence of correlations between the methoxy group and the aromatic ring ruled out the ether structures. Meanwhile, a ^3^*J* coupling observed between the methyl group and the carbonyl (Figure 2) enabled us to conclusively elucidate **2** as the methyl ester that we named caudiquinol.

## 3. Discussion

**Figure 3:**
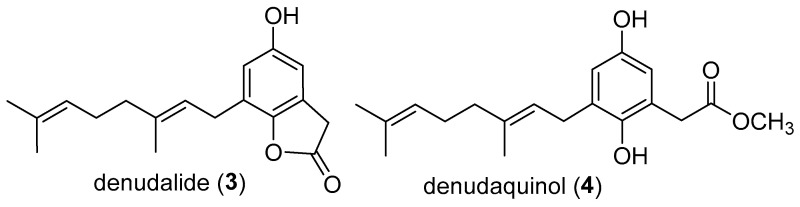
The geranyl meroterpenoids denudalide (**3**) and denudaquinol (**4**).

The lactone versus methyl ester relationship between meroterpenoids **1** and **2** was precedented by a shorter C10 geranyl sidechain by the pair of natural products denudalide (**3**) and denudaquinol (**4**) (Figure 3) isolated from fruits of *Magnolia denudata* of the Magnoliaceae family [9]. Although denudalide could give rise to denudaquinol, in principle, by methanolic hydrolysis, the authors could not demonstrate this conversion in the laboratory. Similarly, given our mild HPLC conditions (aq MeOH; pH 7; rt), we believe that caudiquinol is an authentic natural product.

**Figure 4:**
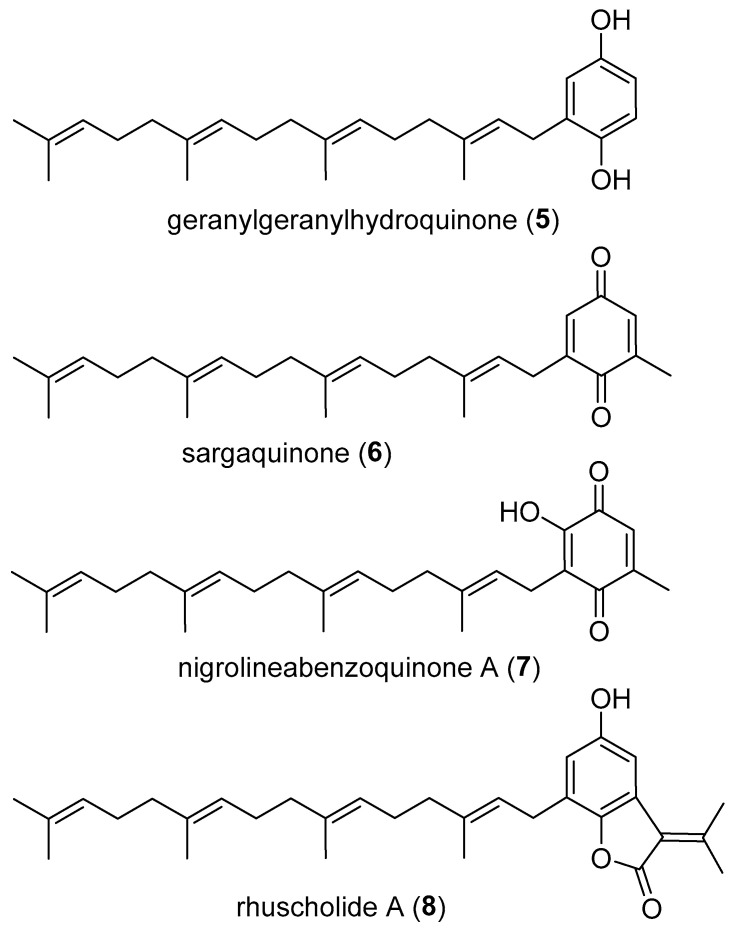
Plant meroterpenoids, other than **1** and **2**, with an intact geranylgeranyl sidechain.

Meroterpenoids that contain units larger than the simple C5 prenyl (dimethylallyl) group typically undergo further transformations, such as oxidation or cyclization, whereas **1**–**4** feature unmodified C10 or C20 sidechains. The discovery of **1**–**4** from four different tree families suggests a common biosynthetic pathway to such meroterpenoids within the plant kingdom, and that congeners with an intermediate C15 sidechain are also likely to be found in nature. Furthermore, in addition to **1** and **2**, we are aware of only four other meroterpenoids (**5**–**8** (Figure 4)) of plant origin with an intact C20 geranylgeranyl unit [7,10,11,12].

Purified **1** and **2** were inactive against seven Gram-positive bacterial strains assayed (MSSA 25923, methicillin-resistant *S. aureus* (MRSA) 13373, SA XU212, SA 1199B, SA RN4220, *Enterococcus faecalis* 12967, and *E. faecalis* 51299) with MIC values of >250 µM or against the A549 lung cancer cell line at 100 µM. We did not have access to the HIV virus or the SFME cell line against which **1**, **3**, and **4** were reported to be active. We conclude the original antibiotic activity of the crude extract arises from other components within the mixture.

## 4. Materials and Methods

General experimental procedures: Vacuum liquid chromatography (VLC) was performed using dry silica gel 60 PF_254+366_ (Merck, London, UK). LC-QToF-MS/MS data were acquired using an Agilent (Santa Clara, CA, USA) 6546 Quadrupole/Time-of-Flight (Q-ToF) mass spectrometer with 1290 UHPLC, equipped with a Phenomenex Kintex C_18_ column (100 × 2.1 mm, 2.6 μm, 100 Å) using deionized H_2_O/MeCN (95:5 to 5:95 gradient with 0.1% HCO_2_H over 5 min 50 s) eluent mixture. Preparative HPLC was performed using a recycling LaboACE LC-5060 series HPLC instrument fitted with a C18 column (20 × 500 mm, 10 μm, 120 Å) (JAI, Tokyo, Japan) and a flow rate of 10 mL/min. One- and two-dimensional (1D and 2D) NMR spectra were recorded with a 500 MHz spectrometer (Bruker, Billerica, MA, USA) using a chloroform-d solvent. The spectra were processed using the MestReNova 14.1 software. UV–visible absorption spectra were recorded with a Perkin Elmer (Shelton, CT, USA) UV/Vis Lambda 365 spectrophotometer. IR absorbance spectra were recorded with a Perkin Elmer FT-IR System Spectrum BX.

Plant material: Leaves of *Garcinia caudiculata* Ridl. were collected at Lundu, Sarawak, Malaysia (1°37′15″ N, 109°45′57″ E). The samples were taxonomically identified by one of the authors, Stephen Ping Teo, and deposited as a voucher specimen STP86 at the Forest Herbarium (SAR), the Forest Department Sarawak. The leaves were air-dried and ground into a fine powder before storage. 

Extraction and isolation: The dry, powdered leaves (100 g) were extracted by macerating them with CH_2_Cl_2_ at room temperature (1 L × 3 times for 24 h each). The extracts were filtered, and the supernatant was concentrated under reduced pressure at 40 °C to obtain the combined crude extract (10 g). Half of the crude extract (5 g) was separated using VLC via silica gel into 16 fractions using a mixture of two solvents (hexane and ethyl acetate) of increasing polarity. Of these, fractions 10 (554 mg) and 11 (459 mg), eluted with 50% and 30% hexane, respectively, were purified by preparative HPLC, using 1 mL volume injections. Fraction 10 was injected at 10 mg/mL and was eluted using 100% MeOH to yield compound **1** (7.4 mg). Fraction 11 was injected at 12 mg/mL and eluted using a gradient of deionized H_2_O/MeOH of 20%:80% for 10 min followed by a linear gradient reaching 100% MeOH at 15 min to yield caudiquinol **2** (6.0 mg).

*Methyl 3-[(2E, 6E, 10E, 14E)-3,7,11,15-tetramethyl-2,6,10,14-hexadecatetraen-1-yl]-2,5-dihydroxybenzeneacetate (caudiquinol* **2***)*. 6.0 mg; yellow oil; UV λ_max_ (MeOH): 220, 232, and 294 nm; IR: 3387, 2914, 1714, and 1435 cm^−1^; m/z 455.316 [M + H]^+^ (calcd. for C_29_H_43_O_4_; 455.316; Δ = 0 ppm); ^1^H NMR (500 MHz): δ 6.50 (d, *J* = 3.1 Hz, 1H), 6.41 (d, *J* = 3.1 Hz, 1H), 5.20–5.25 (m, 1H), 5.00–5.09 (m, 3H), 3.66 (s, 3H), 3.53 (s, 2H), 3.27 (d, *J* = 7.0 Hz, 2H), 1.90–2.02 (m, 12H), 1.66 (s, 3H), 1.61 (s, 3H), and 1.53 (s, 9H). ^13^C NMR (126 MHz): δ 173.9, 149.0, 147.0, 138.0, 135.3, 135.0, 131.3, 130.6, 124.4, 124.2, 123.9, 121.5, 121.6, 115.9, 115.0, 52.5, 39.75, 39.73, 39.69, 37.2, 29.2, 26.7, 26.6, 26.5, 25.6, 17.8, 16.3, 16.20, and 16.15.

## Figures and Tables

**Table 1:** ^1^H NMR (500 MHz) and ^13^C NMR (126 MHz) data for compound **2** in CDCl_3_.

Position	δ_C_, Type	δ_H_, Type
1	147.0, C	
2	121.5, C	
3	115.0, CH	6.50, d
4	149.0, C	
5	115.9, CH	6.41, d
6	131.3, C	
7	29.2, CH_2_	3.27, d
8	121.6, CH	5.20–5.25, m
9	138.0, C	
10	37.2, CH_2_	1.90–2.02, m
11	26.5, CH_2_	1.90–2.02, m
12	123.9, CH	5.00–5.09, m
13	135.3, C	
14	39.69, CH_2_	1.90–2.02, m
15	26.6, CH_2_	1.90–2.02, m
16	124.2, CH	5.00–5.09, m
17	135.0, C	
18	39.73, CH_2_	1.90–2.02, m
19	26.7, CH_2_	1.90–2.02, m
20	124.4, CH	5.00–5.09, m
21	130.6, C	
22	25.6, CH_3_	1.54, s
1′	39.75, CH_2_	3.53, s
2′	173.9, C	
3′	52.5, CH_3_	3.66, s
1″	16.3, CH_3_	1.66, s
2″	16.2, CH_3_	1.61, s
3‴	16.2, CH_3_	1.54, s
4″	17.8, CH_3_	1.54, s

## Data Availability

The data are contained within this article and the Supplementary Materials.

## Supplementary Materials

The following supporting information can be downloaded at https://www.mdpi.com/article/10.3390/molecules29153613/s1. Characterization data for **1** and **2** comprising NMR, MS, IR, and UV spectra.
