# Supplementary material for: Caudiquinol: A Meroterpenoid with an Intact C20 Geranylgeranyl Chain Isolated from Garcinia caudiculata
Source: Molecules. 2024 Jul 31;29(15):3613. doi: 10.3390/molecules29153613 (PMC11314301; doi:10.3390/molecules29153613)
Supplement: Supplementary file 1 [file molecules-29-03613-s001.zip › molecules-3088119-supplementary.pdf]

Supplementary materials

Caudiquinol: A Meroterpenoid with an Intact C20 Geranylgeranyl Chain Isolated from *Garcinia caudiculata*

Maya Valmiki [1], Stephen Ping Teo [2], Pedro Ernesto de Resende [3], Simon Gibbons [4] and A. Ganesan [1,*]

[1] School of Pharmacy, University of East Anglia, Norwich Research Park, Norwich NR4 7TJ, UK; m.valmiki@uea.ac.uk
[2] Forest Department Sarawak Headquarters, Medan Raya, Petra Jaya, 93050 Kuching, Sarawak, Malaysia; stephetp@sarawak.gov.my
[3] The John Innes Centre, Norwich Research Park, Norwich NR4 7UH, UK; pedro.de-resende@jic.ac.uk
[4] Natural and Medical Sciences Research Center, University of Nizwa, PC 616, Birkat Al-Mauz, Nizwa P.O. Box 33, Oman; simon@unizwa.edu.om
* Correspondence: a.ganesan@uea.ac.uk

Table of Contents

Characterization data of **1** 1
Figure S1 ^{1}H NMR spectrum of **1** 1
Figure S2 ^{13}C NMR spectrum of **1** 2
Figure S3 COSY NMR spectrum of **1** 3
Figure S4 DEPT NMR spectrum of **1** 4
Figure S5 HSQC NMR spectrum of **1** 5
Figure S6 HMBC NMR spectrum of **1** 6
Figure S7 ESI-MS spectrum of **1** 6
Figure S8 IR spectrum of **1** 7
Figure S9 ^{1}H NMR spectrum of **2** 7
Figure S10 ^{13}C NMR spectrum of **2** 8
Figure S11 COSY NMR spectrum of **2** 9
Figure S12 DEPT NMR spectrum of **2** 10
Figure S13 HSQC NMR spectrum of **2** 11
Figure S14 HMBC NMR spectrum of **2** 12
Figure S15 ESI-MS spectrum of **2** 12
Figure S16 IR spectrum of **2** 13
Figure S17 UV spectrum of **1** and **2** 13

5-hydroxy-7-(3,7,11,15-tetramethylhexadeca-2,6,10,11-tetraenyl)-2(3H)-benzofuranone (**1**). 7.4 mg; Dark yellow oil; UV λ_{max} (MeOH) 222, 232 and 299 nm; IR ν_{max} 3390, 2918, 1713, 1440 cm^{-1}; m/z 423.289 [M+H]$^+$ (calcd. for $C_{28}H_{39}O_3$, 423.2899, Δ= - 2.2 ppm); ^{1}H NMR (500 MHz) δ 6.54 (d, *J* = 2.7 Hz, 1H), 6.51 (d, *J* = 2.8 Hz, 1H), 5.19 – 5.26 (m, 1H), 5.00 - 5.09 (m, 3H), 3.62 (s, 3H), 3.53 (s, 2H) 3.26 (d, *J* = 7.2 Hz, 2H), 1.89 - 2.05 (m, 12H), 1.65 (d, *J* = 10.3 Hz, 3H), 1.61 (s, 3H), 1.54 (s, 9H); ^{13}C NMR (126 MHz) δ 173.5, 151.2, 145.8, 136.7, 134.2, 134.0, 130.3, 124.9, 123.4, 123.2, 122.9, 122.6, 119.4, 114.3, 108.2, 38.70, 39.67 (2C), 32.9, 26.7, 25.7, 25.6, 25.4, 24.7, 16.7, 15.20, 15.04, 14.99.

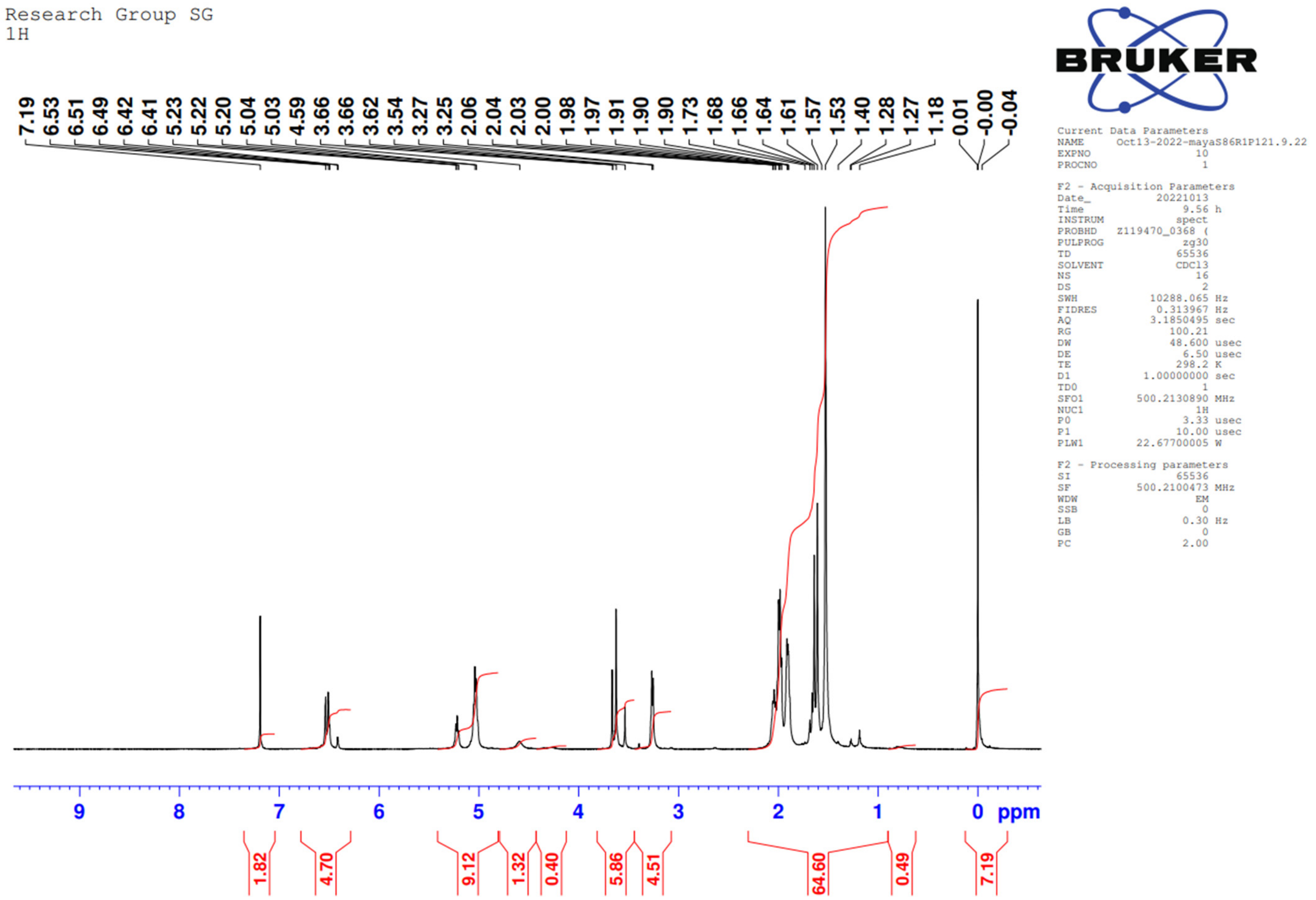

Figure S1 ^{1}H NMR spectrum of **1** in $CDCl_3$ (500 MHz)

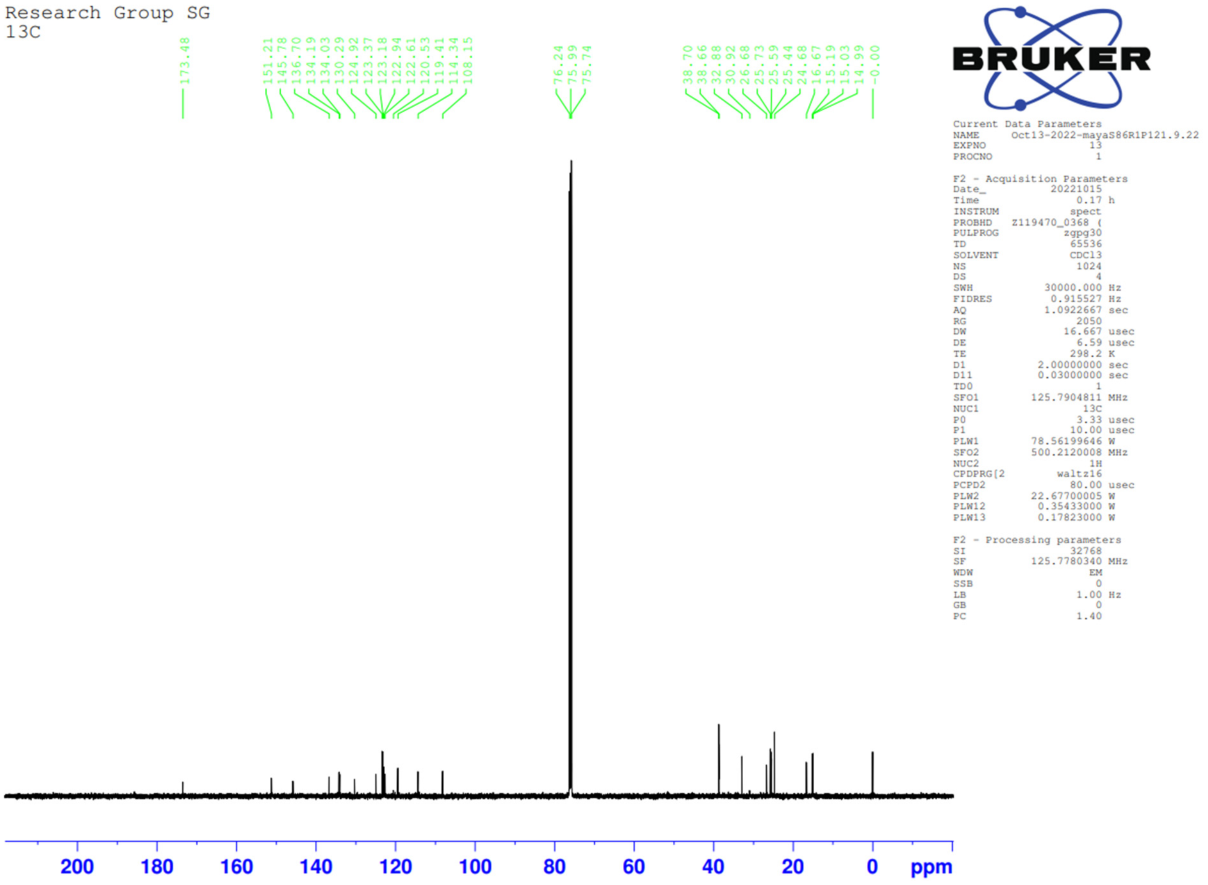

Figure S2 ^{13}C NMR spectrum of **1** in $CDCl_3$ (500 MHz)

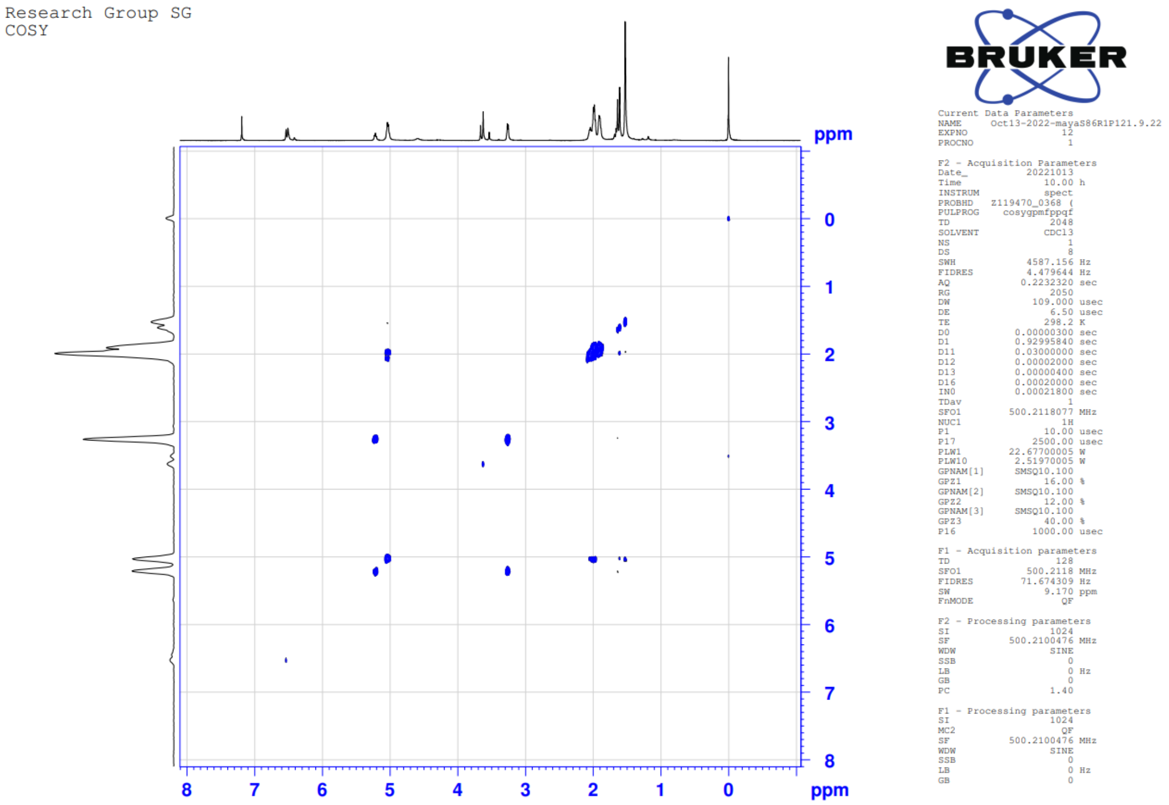

Figure S3 COSY NMR spectrum of **1** in $CDCl_3$ (500 MHz)

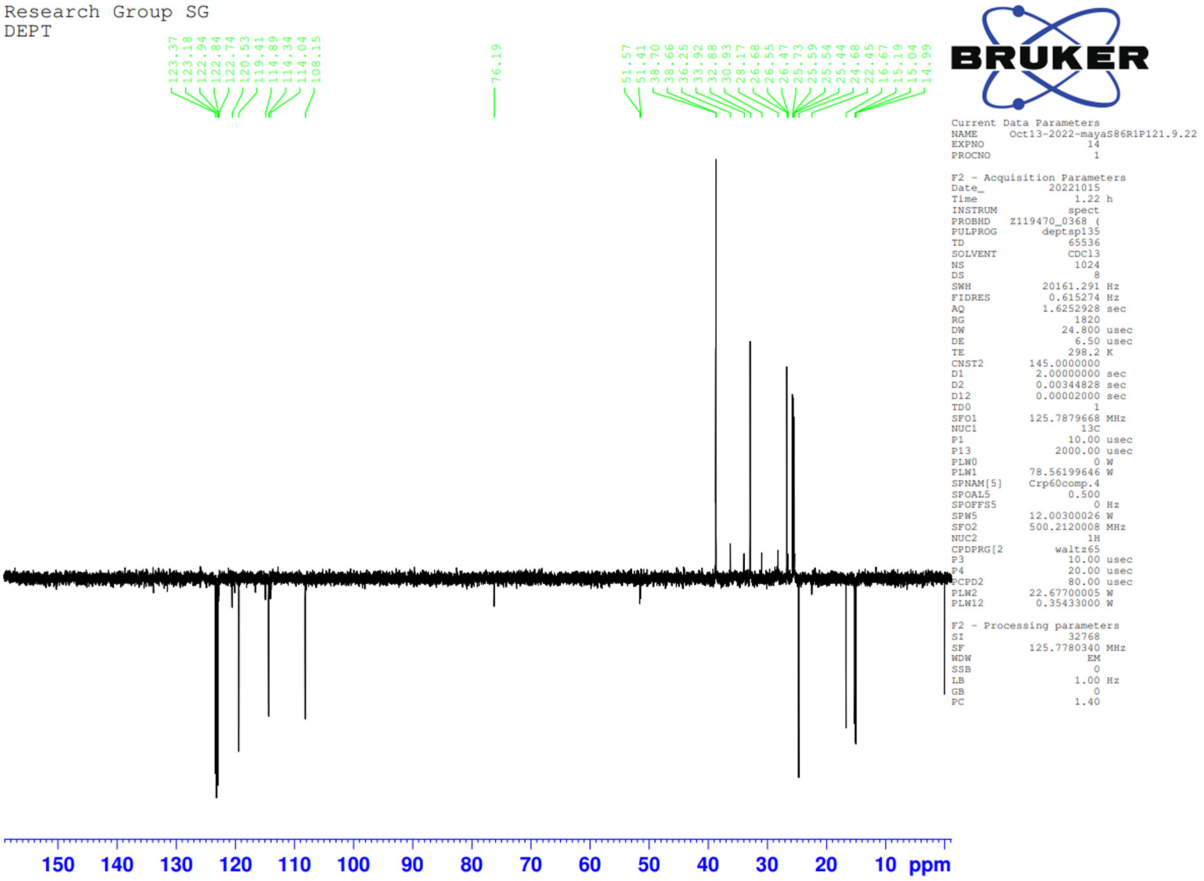

Figure S4 DEPT NMR spectrum of **1** in $CDCl_3$ (500 MHz)

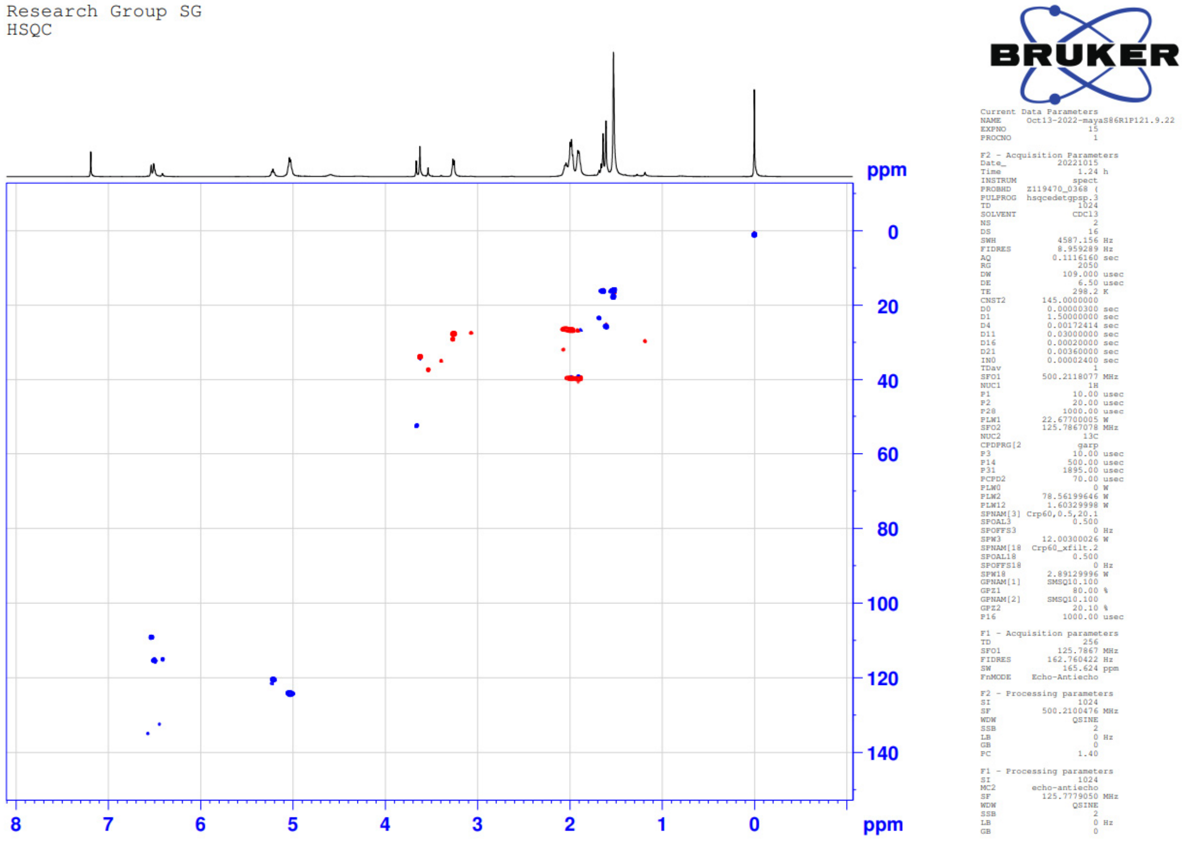

Figure S5 HSQC NMR spectrum of **1** in $CDCl_3$ (500 MHz)

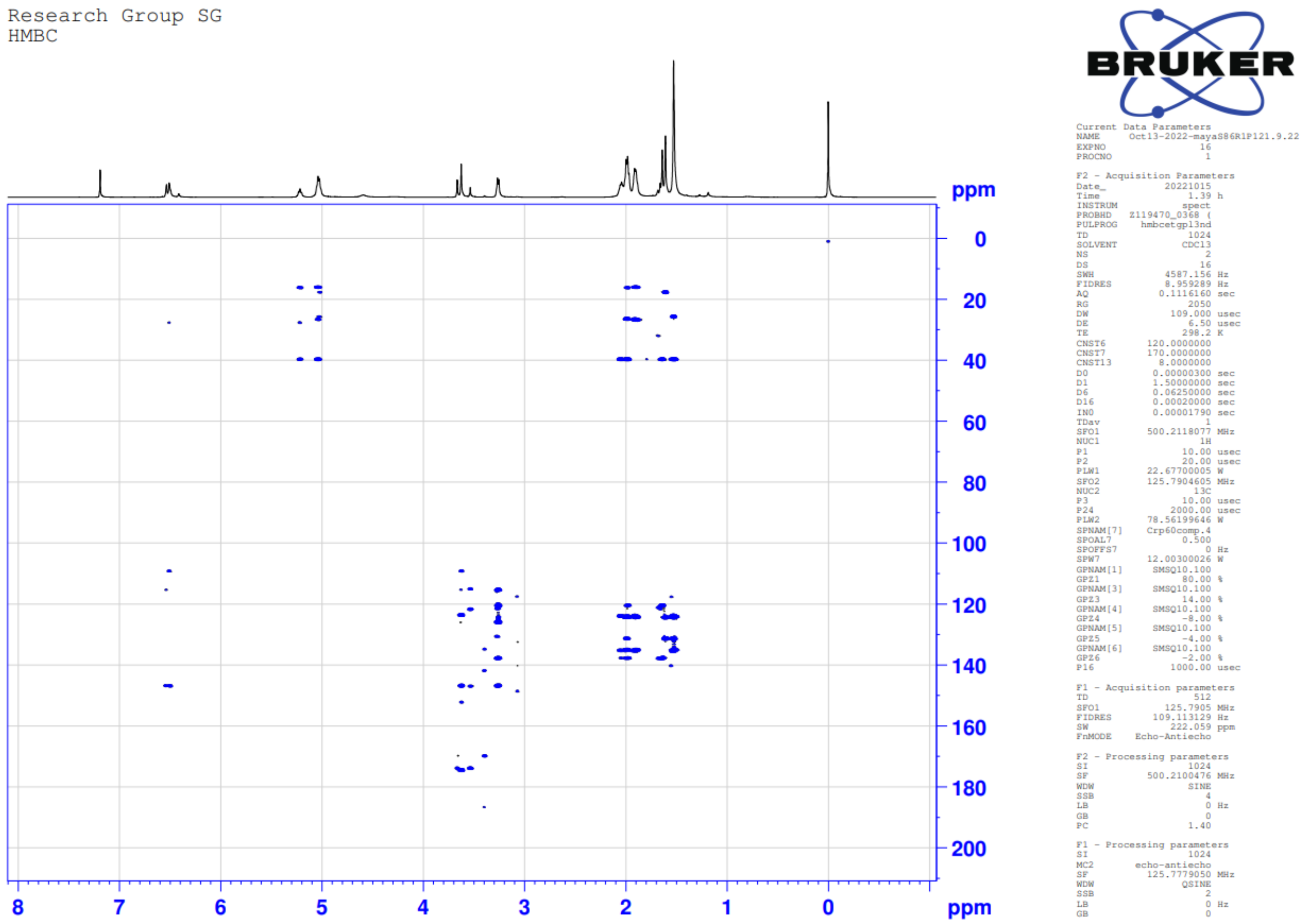

Figure S6 HMBC NMR spectrum of **1** in $CDCl_3$ (500 MHz)

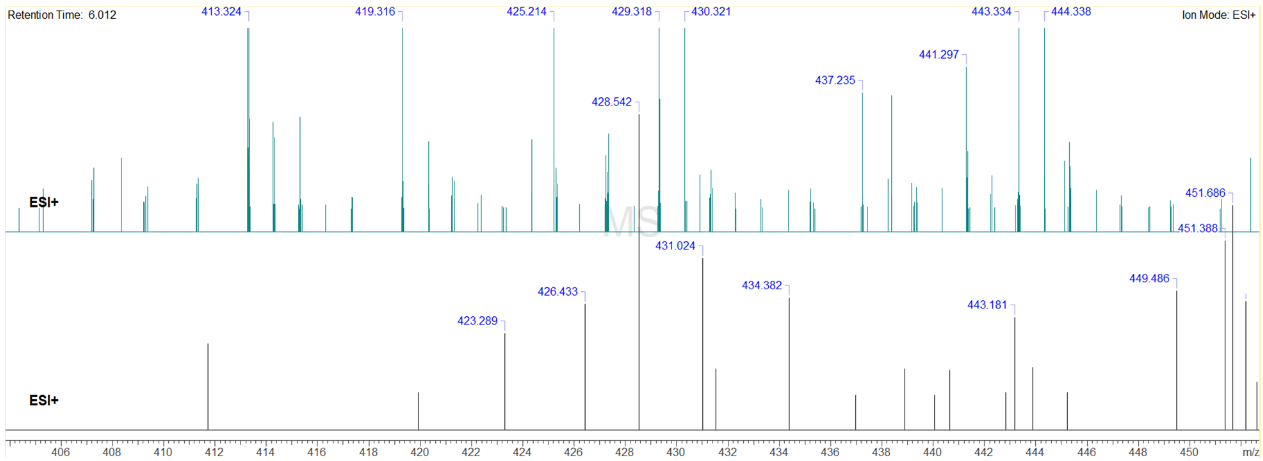

Figure S7 ESI-MS spectrum of **1** showing *m/z* 423.289 $[M+H]^+$

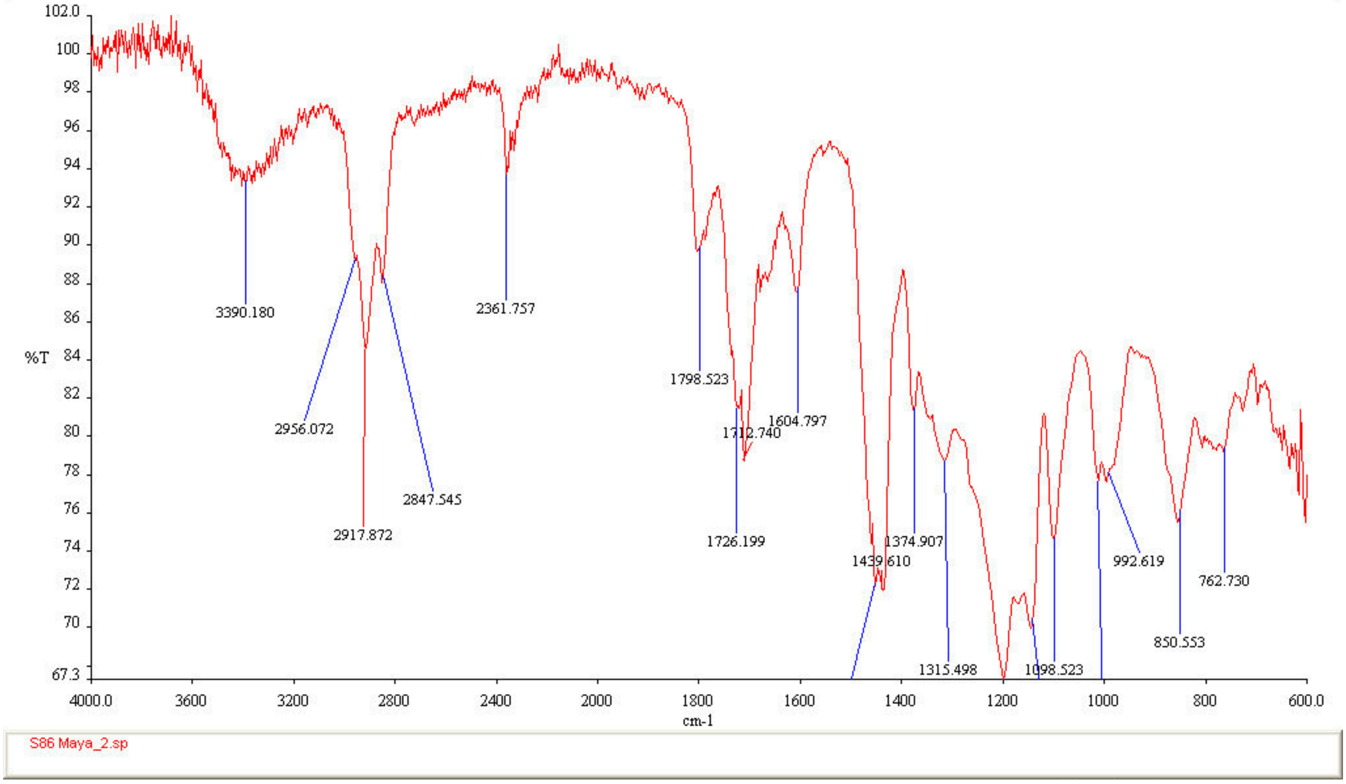

Figure S8 IR spectrum of **1**

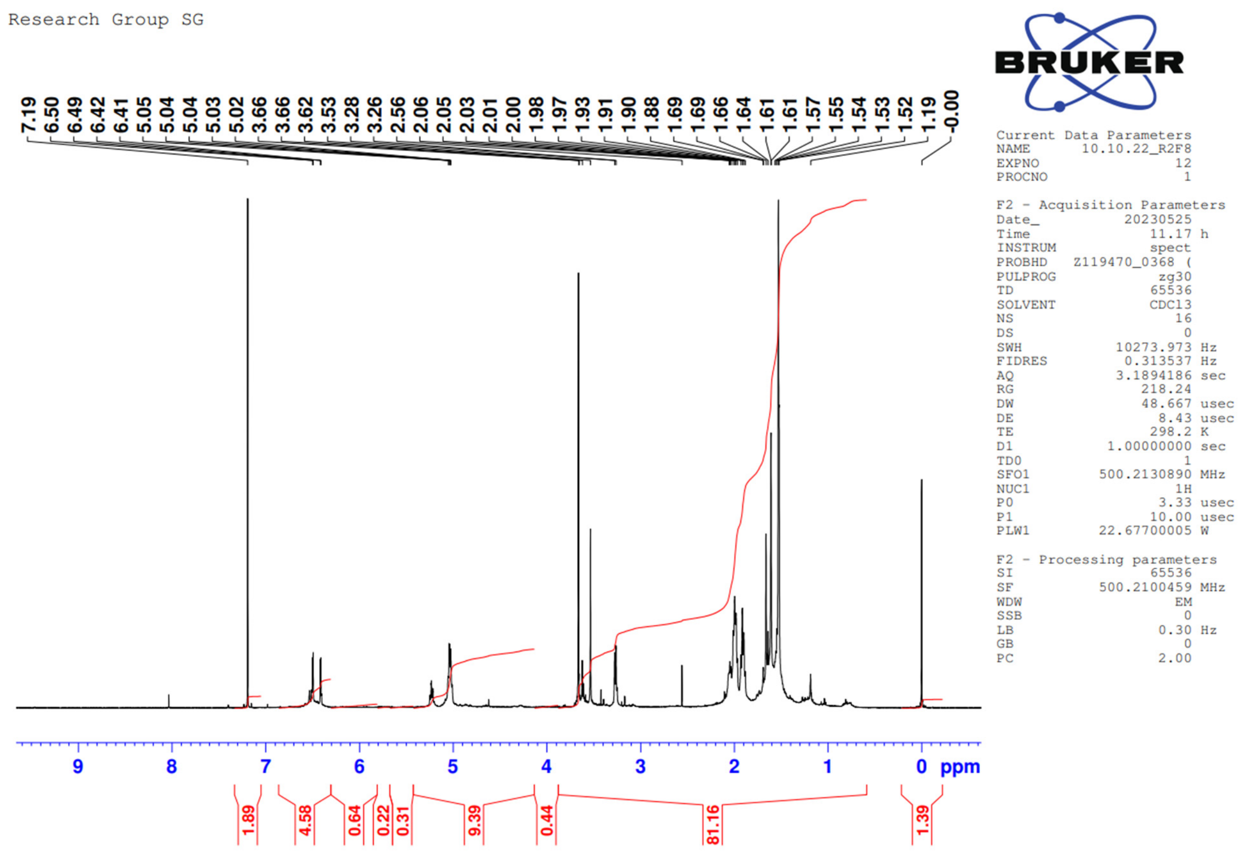

Figure S9 ^{1}H NMR spectrum of **2** in $CDCl_3$ (500 MHz)

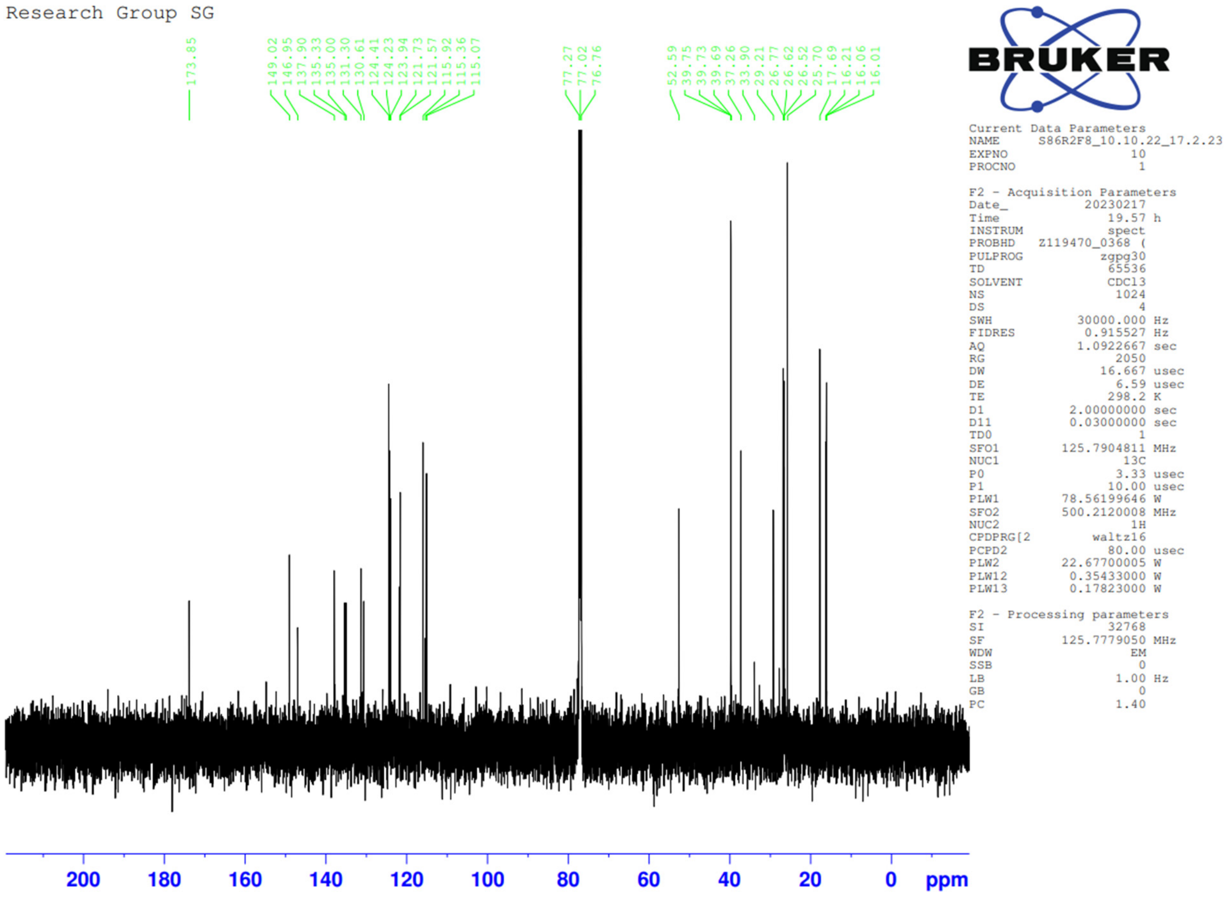

Figure S10 ^{13}C NMR spectrum of **2** in $CDCl_3$ (500 MHz)

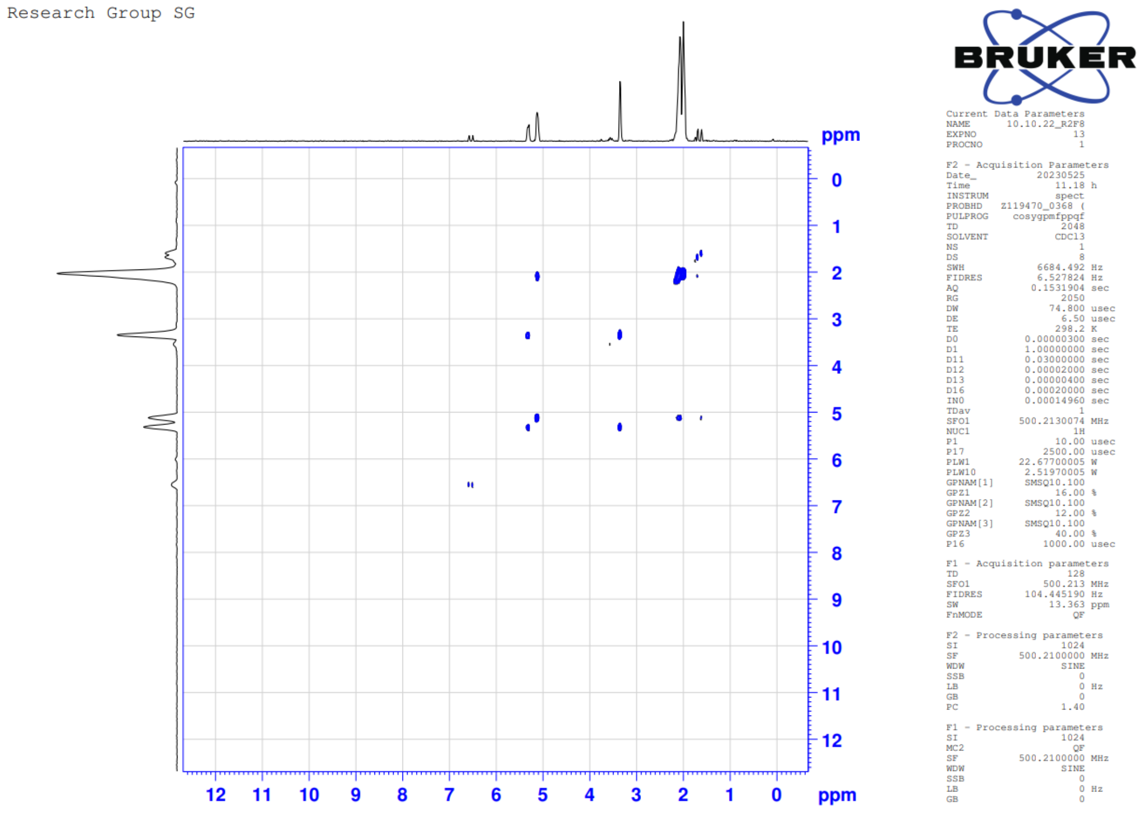

Figure S11 COSY NMR spectrum of **2** in $CDCl_3$ (500 MHz)

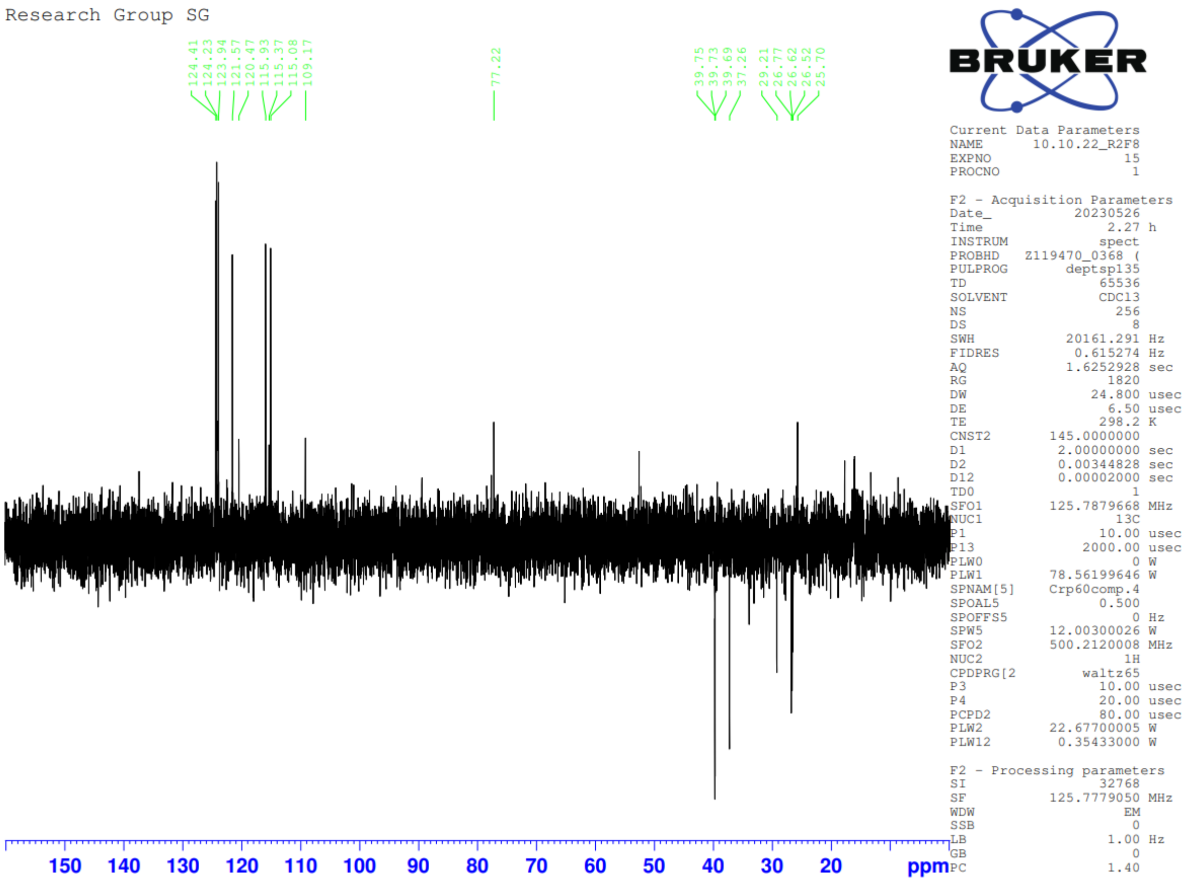

Figure S12 DEPT NMR spectrum of **2** in $CDCl_3$ (500 MHz)

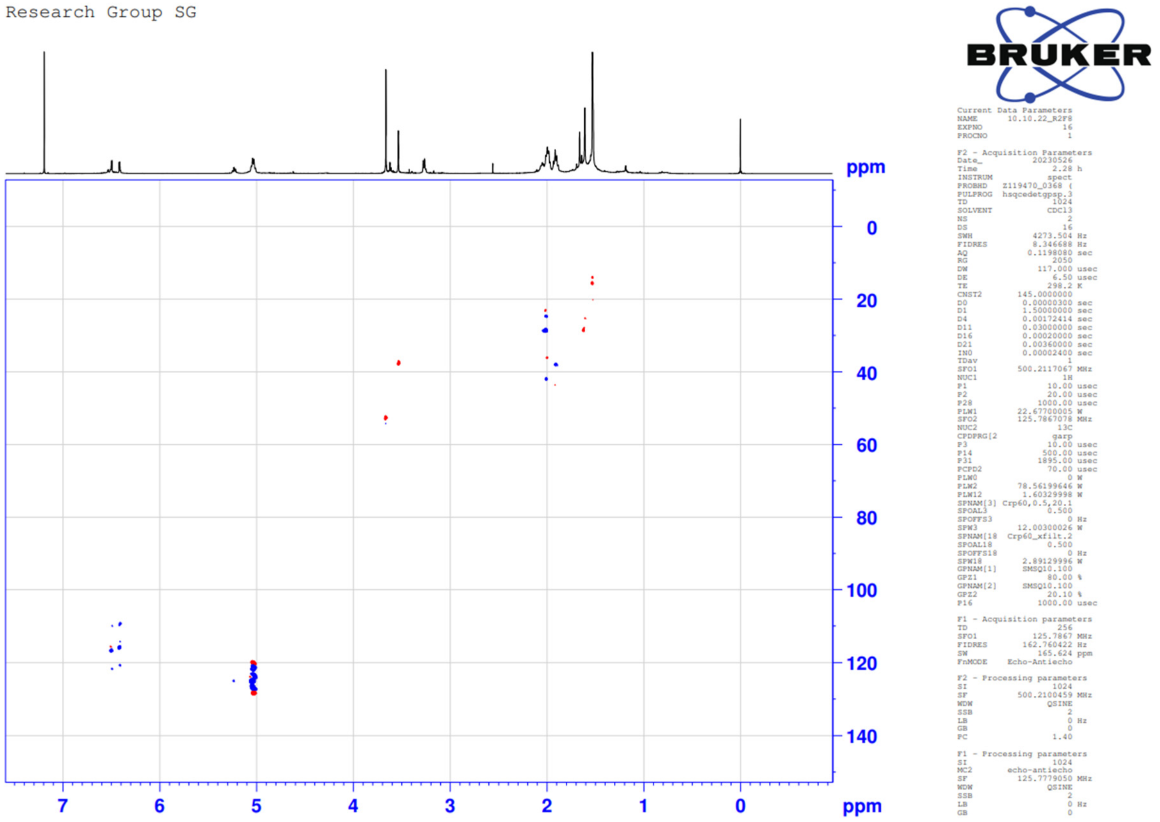

Figure S13 HSQC NMR spectrum of **2** in $CDCl_3$ (500 MHz)

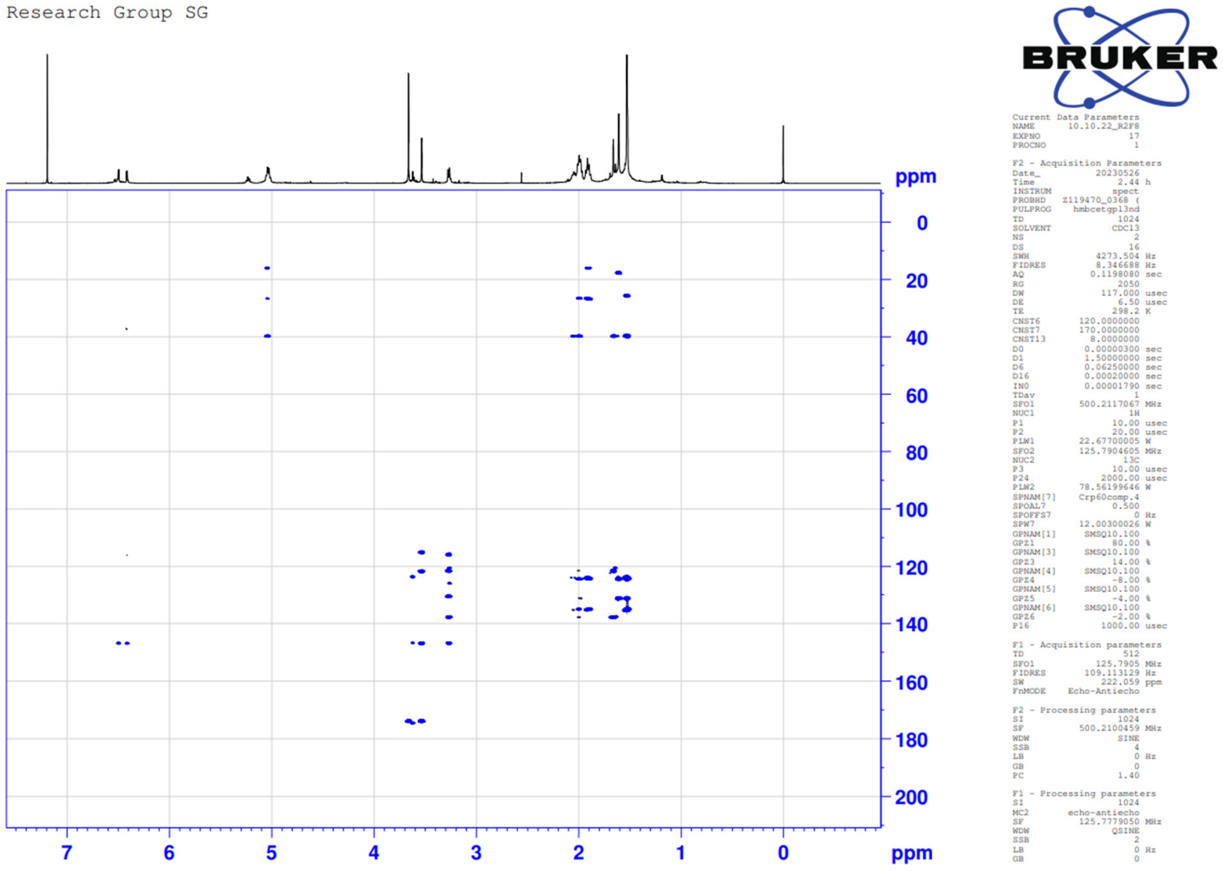

Figure S14 HMBC NMR spectrum of **2** in $CDCl_3$ (500 MHz)

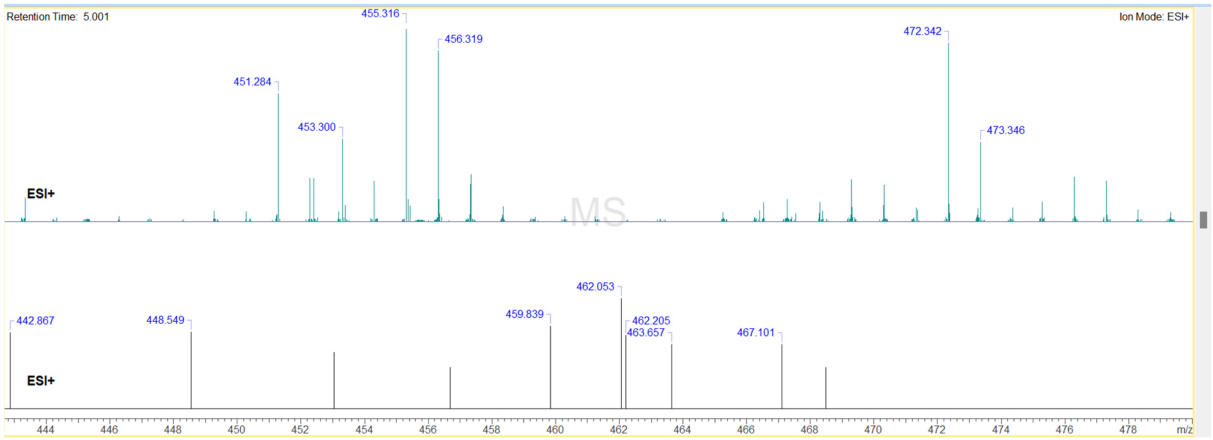

Figure S15 ESI-MS spectrum of **2** showing *m/z* 455.316 $[M+H]^+$

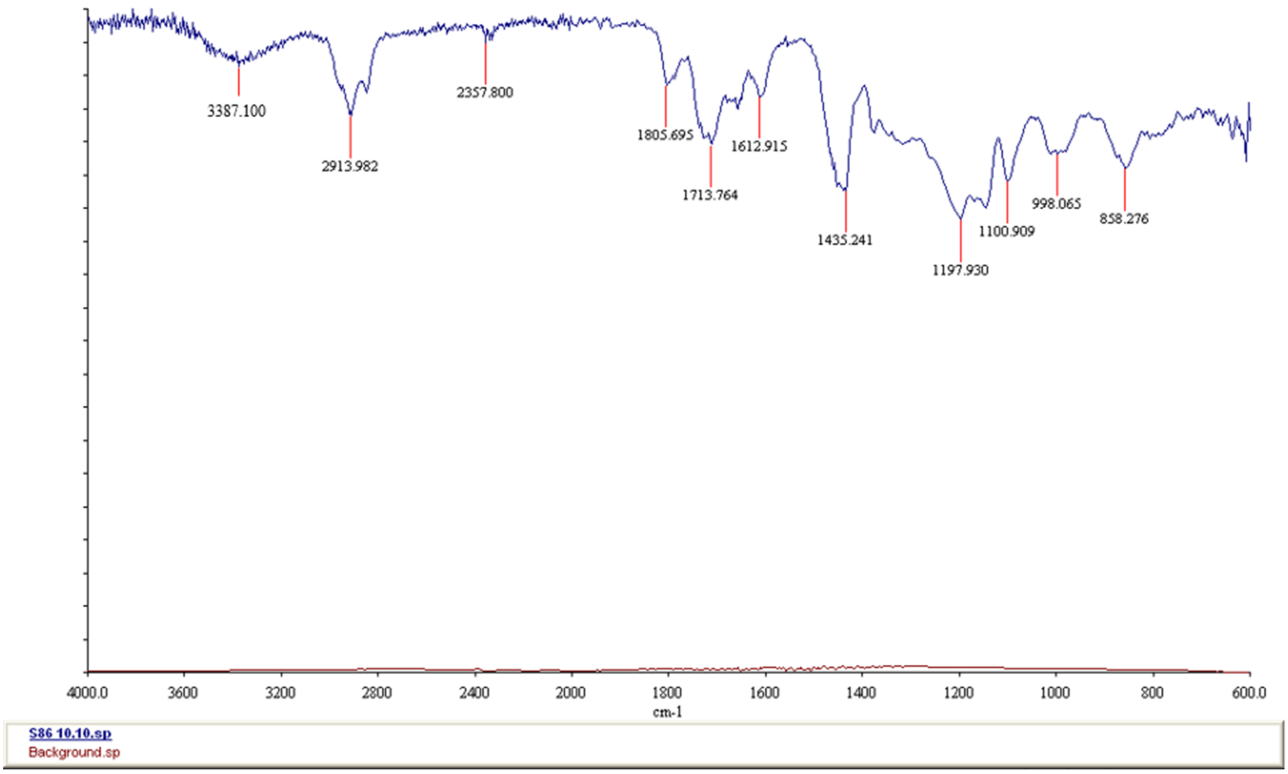

Figure S16 IR spectrum of **2**

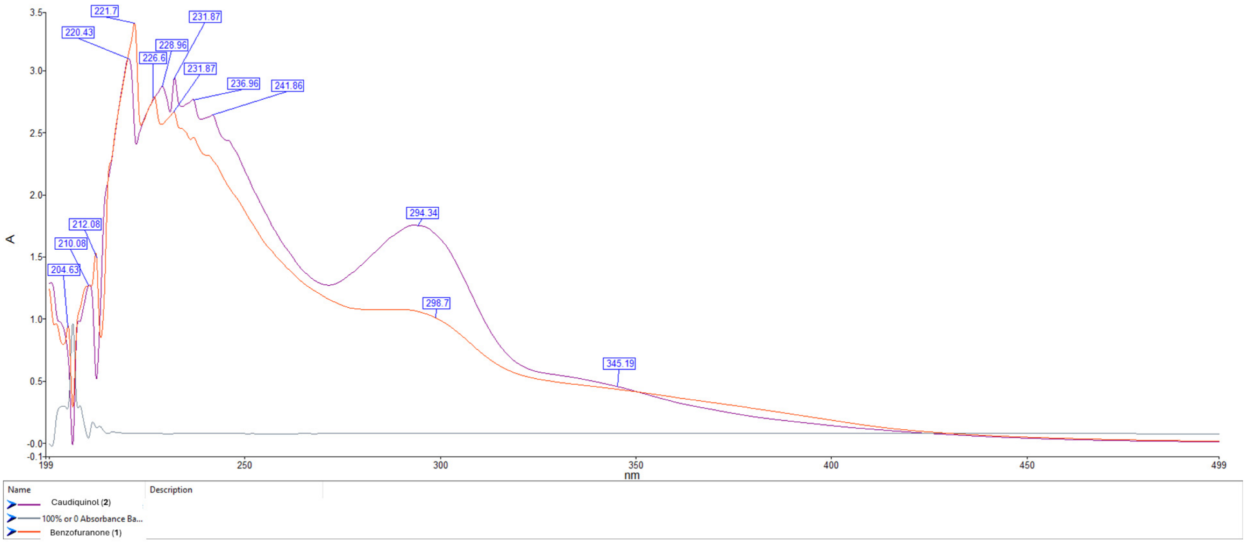

Figure S17 UV spectrum of **1** and **2**
